# Simvastatin Does Not Affect Nitric Oxide Generation Increased by Sesame Oil in Obese Zucker Rats

**DOI:** 10.1155/2018/5413423

**Published:** 2018-08-30

**Authors:** Martina Cebova, Radoslava Rehakova, Michaela Kosutova, Olga Pechanova

**Affiliations:** Institute of Normal and Pathological Physiology, Centre of Experimental Medicine, Slovak Academy of Sciences, Sienkiewiczova 1, 813 71 Bratislava, Slovakia

## Abstract

Current treatments for cardiovascular and obesity-associated diseases, such as statin therapy, may be associated with several side effects. Products from food sources with polyphenolic compounds may represent promising agents in the treatment of cardiovascular and metabolic diseases with minimal side effects. Thus, we aimed to study the effect of sesame oil and simvastatin treatment on plasma lipid profile, nitric oxide generation, and oxidative load in obese Zucker rats. 12-week-old male Zucker rats were divided into the control and sesame oil- (1.25 ml/kg/day) treated Zucker lean groups, the control and sesame oil (1.25 ml/kg/day), or simvastatin (15 mg/kg/day) together with sesame oil-treated Zucker fa/fa groups, *n* = 6 in each group. The treatment lasted for 6 weeks. Sesame oil composition and plasma lipid profile were analyzed. Nitric oxide synthase (NOS) activity, endothelial NOS (eNOS), phosphorylated eNOS, and inducible NOS (iNOS) protein expressions were determined in the left ventricle and aorta. Oxidative load, measured as conjugated diene (CD) and thiobarbituric acid reactive substance (TBARS) concentrations, was detected in the liver. Neither sesame oil nor cotreatment with simvastatin affected plasma lipid profile in Zucker fa/fa rats. Sesame oil and similarly cotreatment with simvastatin markedly increased NOS activity and phosphorylated eNOS protein expressions in the left ventricle and aorta of Zucker fa/fa rats. There were no changes in eNOS and iNOS protein expressions within the groups and tissues investigated. Hepatic CD concentration was higher in Zucker fa/fa comparing Zucker lean rats, and sesame oil treatment decreased it significantly. Interestingly, this decrease was not seen after cotreatment with simvastatin. In conclusion, phosphorylation of eNOS and decreased oxidative load may significantly contribute to increase in total NOS activity with potential beneficial properties. Interestingly, simvastatin did not affect NO generation already increased by sesame oil in obese Zucker rats.

## 1. Introduction

In the modern society, metabolic syndrome clustering obesity, hypertension, dyslipidemia, and hyperglycemia represent one of the major causes of atherosclerosis, heart failure, and stroke. The average prevalence of metabolic syndrome is 31% and is associated with a twofold increase in the risk of coronary heart disease and cerebrovascular disease and a 1.5-fold increase in the risk of all-cause mortality [1]. Each component of metabolic syndrome is an independent risk factor for cardiovascular disease, and a combination of them elevates rates and severity of different pathophysiological conditions including microvascular dysfunction, coronary atherosclerosis, cardiac dysfunction, myocardial infarction, and heart failure [2, 3]. Current treatments for cardiovascular and obesity-associated diseases, such as statin therapy, may be associated with considerable residual risk and several side effects in some patients [4]. Research focused on the identification of alternative pharmaceutical agents for the treatment of cardiovascular diseases has been relatively disappointing, especially on the clinical level. Recently, products from food sources with polyphenolic compounds represent promising agents in the treatment of cardiovascular and metabolic diseases [4–8].

The cardioprotective effects of polyphenolic compounds have been linked mainly to its antioxidant properties; however, recent findings attribute its antiatherosclerotic potential to modulation of different signaling pathways [9]. Emerging data suggest that polyphenols can regulate cellular lipid metabolism, platelet function, and vascular function, especially endothelial function, which represent primary conditions for atherosclerotic plaque formation and development [5, 10].

Sesame, with several polyphenolic ligands and high oil content (50–60%) [11], becomes a promising tool for treatment of cardiovascular diseases. Sesame oil is an excellent source of unsaturated fatty acids consist from oleic acid (37%) and linoleic fatty acid (46%) [12]. Sesame oil also contains an amount of bioactive components such as tocopherols, polyphenols, flavonoids, phenolic ligands, sesamin, and sesamolin. All of them are considered to be protective [13, 14] acting as antioxidants, antihypertensives, anti-inflammatory, and cardioprotective substances [15–17]. By reducing 3-hydroxy-3-methylglutaryl-CoA (HMG-CoA) reductase activity, sesamin could also potentially reduce LDL levels in a similar manner as statin drugs without side effects [18]. Similarly, different other polyphenols, also those including in the sesame, may improve vasoactivity, endothelial function, and nitric oxide (NO) production.

Thus, the aim of our study was to evaluate the effect of the sesame oil and simvastatin treatment on plasma lipid profile and nitric oxide generation in the left ventricle and aorta, as well as on hepatic oxidative load in obese Zucker rats.

## 2. Materials and Methods

### 2.1. Chemicals

Most of the chemicals and reagents were obtained from Sigma-Aldrich; when not, the company is indicated.

### 2.2. Animals and Treatment

All procedures and experimental protocols were performed in accordance with institutional guidelines and were approved by the State Veterinary and Food Administration of the Slovak Republic (Ro-1998/15-221) and by an ethical committee according to the European Convention for the Protection of Vertebrate Animals used for Experimental and other Scientific Purposes, Directive 2010/63/EU of the European Parliament. Male lean Zucker and obese Zucker fa−/fa− rats were obtained from Charles River, USA. All rats used in the study were born in an accredited breeding establishment. They were housed in groups of 3 animals, under a 12 h light-12 h dark cycle, at a constant humidity (45–65%) and temperature (20–22°C), with free access to standard laboratory rat chow and drinking water.

12-week-old male Zucker rats were divided into the control Zucker lean group, the Zucker lean group treated with sesame oil in the dose of 1.25 ml/kg/day, the Zucker fa/fa control group, the Zucker fa/fa group treated with sesame oil in the dose of 1.25 ml/kg/day, and the Zucker fa/fa group treated with simvastatin (15 mg/kg/day) and sesame oil in the dose of 1.25 ml/kg/day. Each group consists of 6 animals. Treatment was administered via gavage from the 12th week of age for 6 weeks. Daily water consumption was estimated individually for every animal and adjusted, if necessary. All animals were housed at a temperature of 22–24°C and fed with a regular pellet diet ad libitum. Blood pressure was measured noninvasively, using tail-cuff plethysmography weekly. At the end of treatment, the animals were sacrificed, and body weight (BW), heart weight (HW), left kidney weight (LKW), and tibia length (TL) were determined. Relative heart and kidney weights were calculated as HW/tibia or LKW/tibia ratio. Samples of the left ventricle and aorta were used to determine NO synthase (NOS) activity and endothelial NOS (eNOS), phosphorylated eNOS, and inducible NOS (iNOS) protein expressions by Western blot analysis. Lipid profile was analyzed in the plasma and conjugated dienes and thiobarbituric acid reactive substances (TBARS) in the liver.

### 2.3. Sesame Oil and Plasma Lipid Profile

The composition of sesame oil has been commercially determined, and plasma lipid profiles were detected by commercially available kits (Abcam and Crystal Chem, USA).

Briefly, rat blood was collected from the aorta after anesthesia. The levels of triglyceride (TG), total cholesterol (TC) high-density lipoprotein cholesterol (HDL), and low-density lipoprotein cholesterol (LDL) were measured in the plasma using a biochemical enzyme kit (Abcam and Crystal Chem, resp.) according to the protocol by 800TS Absorbance Microplate Reader (BioTek, USA).

### 2.4. Total NOS Activity and Protein Expressions

Total NOS activity was determined in crude homogenates of the left ventricle and aorta by measuring the formation of [^3^H]-L-citrulline from [^3^H]-L-arginine (ARC, Montana, USA) as previously described and slightly modified by Pechánová et al. [19]. [^3^H]-L-citrulline was measured with the Quanta Smart TriCarb Liquid Scintillation Analyzer (Packard Instrument Company, Meriden, CT).

Protein expressions of eNOS and iNOS were determined in the left ventricle and aorta by Western blot analysis. Protein expression of phosphorylated eNOS (peNOS) was determined in the left ventricle only. The samples were probed with polyclonal rabbit, anti-eNOS, anti-peNOS, anti-iNOS, and anti-GAPDH antibodies (Abcam, Cambridge, UK). The intensity of bands was visualized using the enhanced chemiluminescence system (ECL, Amersham, UK), quantified by using ChemiDoc™ Touch Imagine System (Image Lab™ Touch software, Bio-Rad) and normalized to GAPDH bands.

### 2.5. Conjugated Dienes (CD) and Thiobarbituric Acid Reactive Substances (TBARS)

Samples of the liver were homogenized in 15 mmol/l EDTA containing 4% NaCl. Lipids were extracted using 1 : 1 chloroform-methanol mixture. The concentration of CD was estimated as described by Kogure et al. [20]. Chloroform was evaporated in N_2_ atmosphere. After the addition of cyclohexane, the absorbance (NanoDrop 2000 c, UV-Vis spectrophotometer) was determined. The concentration of CD was calculated using the extinction coefficient *ε* = 29,000 l/mol/cm and expressed as *μ*mol per g tissue.

To determine TBARS, 1 ml of 10% tissue homogenates of the liver (in 1.15% KCl in 0.01 mol/l phosphate buffer, pH 7.4) was added to 2 ml of 7.5% trichloroacetic acid and mixed. After centrifugation at 1000*g* for 10 min, 1 ml of the supernatant was added to 0.5 ml of 0.7% 2-thiobarbituric acid and incubated in a hot water bath for 10 min. After cooling, TBARS were measured at 532 nm (NanoDrop 2000 c, UV-Vis spectrophotometer). An extinction coefficient of 156,000 mol^−1^·l·cm^−1^ was used for the calculation of the results.

### 2.6. Statistics

The results are expressed as mean ± SEM. One-way analysis of variance and Duncan test were used for statistical analysis. Values were considered significant with probability value *p* < 0.05 (for both ANOVA and Duncan test).

## 3. Results

### 3.1. General Characteristics

Relative heart and kidney weights did not differ between Zucker lean and Zucker fa/fa-treated or Zucker fa/fa-untreated rats. There were no differences within the groups. Blood pressure did not differ between Zucker lean and Zucker fa/fa rats. Treatment with sesame oil (SO) or SO + simvastatin (SIM) did not affect blood pressure in Zucker rats (data not shown).

### 3.2. Sesame Oil Characterization

The sesame oil contains mainly unsaturated fatty acids; 46.7% from which was linoleic acid and 37.6% oleic acid. Sesame oil had low amount of saturated fatty acid, specifically 1.8% of palmitic acid.

The elements (P, K, and Ca) were detected and their concentrations were determined. Calcium was present at the highest concentration 1.24%, potassium at the concentration of 0.49%, and phosphor 0.20%.

From polyphenolic compounds, the sesame oil contains 4.73% of sesamin and 0.13% of sesamol.

### 3.3. Concentration of Plasma Lipids

Plasma concentrations of total cholesterol, TG, HDL, and LDL were higher in Zucker fa/fa compared with Zucker lean rats. SO treatment increased TG concentration in Zucker lean rats. Neither SO nor SO + SIM affected plasma lipid profile in Zucker fa/fa rats (Table 1).

### 3.4. Total NOS Activity and Protein Expressions

**Figure 1:**
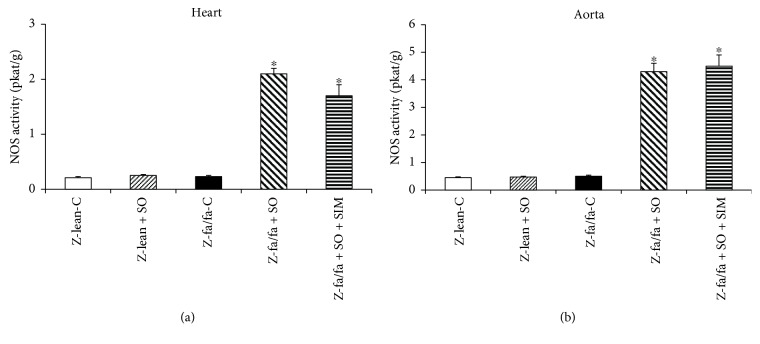
Nitric oxide synthase (NOS) activity in the left ventricle (a) and aorta (b). Data are means ± SEM from 6 animals in each group. Z-lean-C: Zucker lean rats; Z-lean + SO: Zucker lean rats treated with sesame oil; Z-fa/fa-C: control Zucker fatty rats; Z-fa/fa + SO: Zucker fatty rats treated with sesame oil; Z-fa/fa + SO + SIM: Zucker fatty rats treated with sesame oil and simvastatin. ^∗^*p* < 0.05 versus Z-fa/fa-C.

There were no changes in total NOS activity in Zucker lean and Zucker fa/fa rats. SO and SO + SIM cotreatment significantly increased NOS activity in the left ventricle (Figure 1(a)) and aorta (Figure 1(b)) of Zucker fa/fa rats. NOS activity of Zucker lean rats was not changed after SO treatment in both tissues investigated (Figures 1(a) and 1(b)).

**Figure 2:**
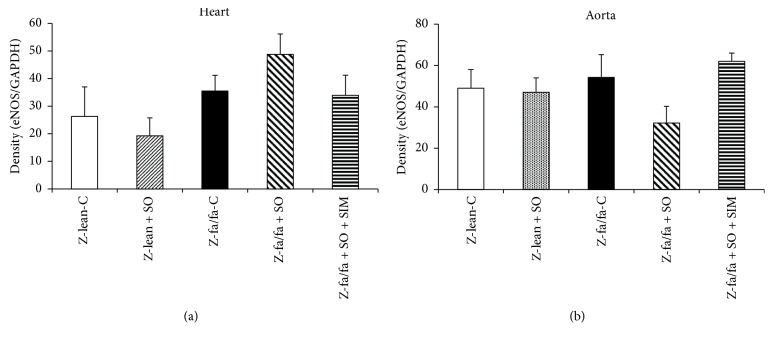
Expression of endothelial NO synthase (eNOS) protein in the left ventricle (a) and aorta (b). Data are means ± SEM from 6 animals in each group. Z-lean-C: Zucker lean rats; Z-lean + SO: Zucker lean rats treated with sesame oil; Z-fa/fa-C: control Zucker fatty rats; Z-fa/fa + SO: Zucker fatty rats treated with sesame oil; Z-fa/fa + SO + SIM: Zucker fatty rats treated with sesame oil and simvastatin.

**Figure 3:**
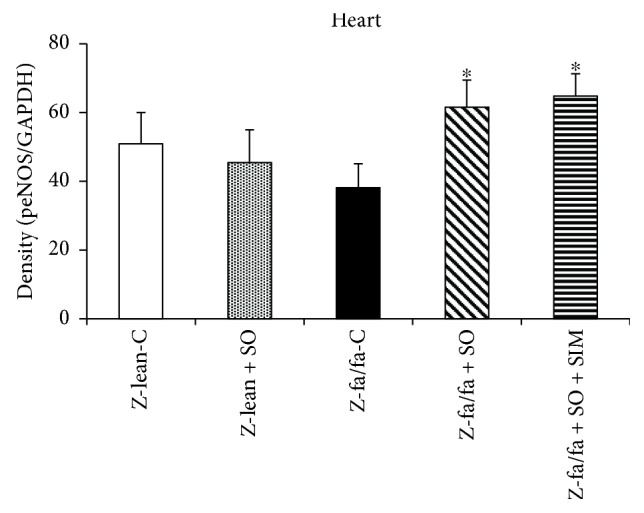
Expression of phosphorylated endothelial NO synthase (peNOS) protein in the left ventricle. Data are means ± SEM from 6 animals in each group. Z-lean-C: Zucker lean rats; Z-lean + SO: Zucker lean rats treated with sesame oil; Z-fa/fa-C: control Zucker fatty rats; Z-fa/fa + SO: Zucker fatty rats treated with sesame oil; Z-fa/fa + SO + SIM: Zucker fatty rats treated with sesame oil and simvastatin. ^∗^*p* < 0.05 versus Z-fa/fa-C.

There were no changes in eNOS protein expressions within the groups and tissues investigated (Figures 2(a) and 2(b)). Expression of phosphorylated eNOS (peNOS) protein in the left ventricle was increased significantly after SO and SO + SIM treatments in Zucker fa/fa rats (Figure 3).

**Figure 4:**
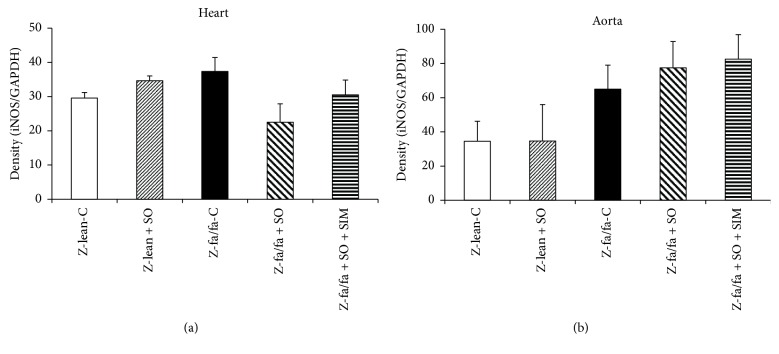
Expression of inducible NO synthase (iNOS) protein in the left ventricle (a) and aorta (b). Data are means ± SEM from 6 animals in each group. Z-lean-C: Zucker lean rats; Z-lean + SO: Zucker lean rats treated with sesame oil; Z-fa/fa-C: control Zucker fatty rats; Z-fa/fa + SO: Zucker fatty rats treated with sesame oil; Z-fa/fa + SO + SIM: Zucker fatty rats treated with sesame oil and simvastatin.

Similarly as in eNOS, there were no changes in iNOS protein expressions within the groups and tissues investigated (Figure 4(a) and 4(b)).

### 3.5. CD and TBARS Concentrations

**Figure 5:**
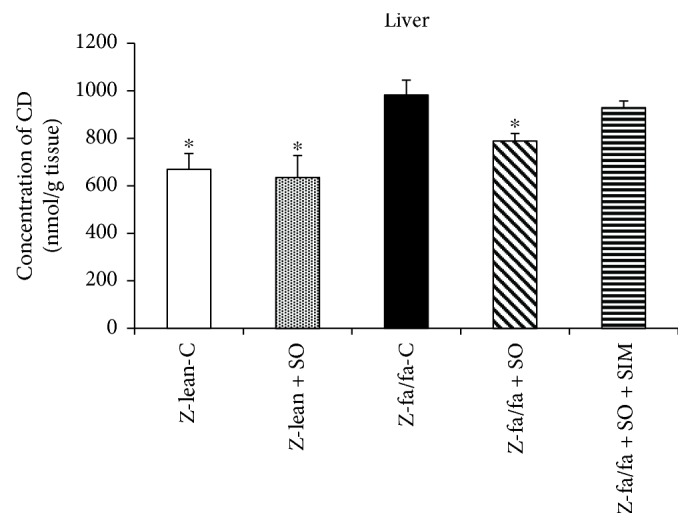
Concentration of conjugated dienes (CD) in the liver. Data are means ± SEM from 6 animals in each group. Z-lean-C: Zucker lean rats; Z-lean + SO: Zucker lean rats treated with sesame oil; Z-fa/fa-C: control Zucker fatty rats; Z-fa/fa + SO: Zucker fatty rats treated with sesame oil; Z-fa/fa + SO + SIM: Zucker fatty rats treated with sesame oil and simvastatin. ^∗^*p* < 0.05 versus Z-fa/fa-C.

**Figure 6:**
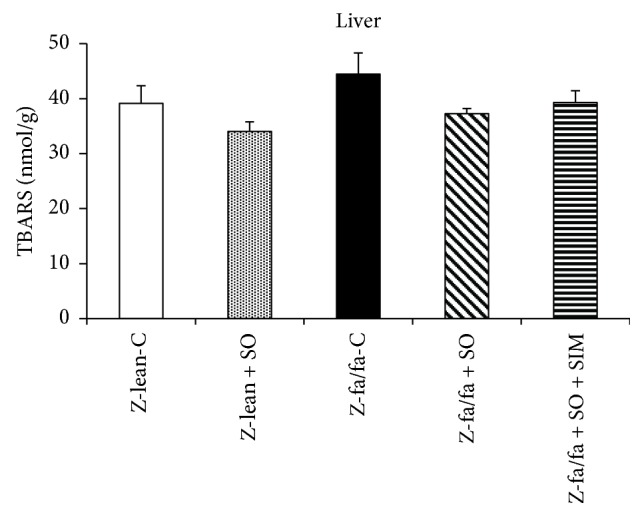
Concentration of thiobarbituric acid reactive substances (TBARS) in the liver. Data are means ± SEM from 6 animals in each group. TBARS: thiobarbituric acid reactive substances; Z-lean-C: Zucker lean rats; Z-lean + SO: Zucker lean rats treated with sesame oil; Z-fa/fa-C: control Zucker fatty rats; Z-fa/fa + SO: Zucker fatty rats treated with sesame oil; Z-fa/fa + SO + SIM: Zucker fatty rats treated with sesame oil and simvastatin.

Hepatic CD concentration was higher in Zucker fa/fa compared with Zucker lean rats, and SO treatment decreased it significantly. Interestingly, this decrease was not seen after SO + SIM treatment (Figure 5). There were no significant changes in TBARS concentration within the groups; however, decreasing trend in TBARS concentration after SO treatment in Zucker fa/fa rats was seen (Figure 6).

## 4. Discussion

Our study documented that neither sesame oil nor sesame oil and simvastatin cotreatment affected plasma lipid profile in Zucker fa/fa rats. Interestingly, sesame oil and similarly sesame oil and simvastatin cotreatment markedly increased NOS activity in the left ventricle and aorta of Zucker fa/fa rats. Phosphorylation of eNOS, decreased lipid peroxidation, and oxidative load may significantly contribute to increased NOS activity in these rats. To the best of our knowledge, this is the first study investigating the effects of sesame oil and simvastatin cotreatment on nitric oxide generation in obese Zucker rats.

Zucker fa/fa rat represents a spontaneous genetic obesity model, which exhibits some components of metabolic syndrome including hyperlipidemia [21]. Hyperlipidemia and other metabolic derangement belong among the major risk factors for cardiovascular diseases [2–4]. Thus, to investigate the effects of sesame oil and simvastatin in conditions of experimental hyperlipidemia, obese Zucker rats were used in our study.

In Zucker fa/fa rats, total cholesterol was increased by 119% in comparison with the lean control. However, simvastatin was not able to decrease cholesterol level significantly. This fact might be explained by a relatively lower dose of simvastatin in our experiment, statin resistance, or insufficient absorption. However, in our parallel experiments (not published data), we were able to see increase of serum bile acids in the presence of simvastatin. Thus, beneficial simvastatin effect may be related to the accelerated transformation of cholesterol into bile acids, which plays important role in the improvement of physiological functions [22]. Sesame oil treatment did not affect plasma lipid profile in Zucker fa/fa rats. In the study of Namayandeh et al. [23] using 48 patients, cholesterol, TG, and LDL were even significantly decreased after sesame oil consumption. Similarly, sesame seed supplementation decreased serum TG, LDL, and lipid peroxidation and increased antioxidant status in hyperlipidemic patients [24].

Recent studies suggested that statins may increase NOS activity under different conditions [25, 26]. Li et al. [26] have reported an increase in eNOS activity after statin treatment through its phosphorylation on serine residue. It has also been documented that phosphorylation of eNOS is necessary for a full activation of eNOS and endothelium-dependent vasorelaxation. The serine/threonine protein kinase Akt functions as an activator of endothelial cell NO production in response to vascular endothelial growth factor and shear flow through its ability to phosphorylate eNOS on serine^1179^ or ^1177^ [27–29]. On the other hand, several reports showed that statins inhibited Akt phosphorylation in response to insulin, especially in cell types other than vascular endothelial cells [30]. Although the reason for this discrepancy remains unclear, it was also reported that statins did not activate Akt in vascular smooth muscle cells or cardiac myocytes [31].

Unfortunately, there are only limited data concerning the effects of sesame oil on NOS activity. Hsu and Parthasarathy [18] found that sesame oil significantly decreased lipid peroxidation but did not increase nitric oxide compared to N-acetyl cysteine. Sharma et al. [32] have found an upregulation of eNOS in rats with cardiometabolic syndrome after sesamol treatment. We have also assumed that polyphenolic compounds involved in sesame oil may increase NOS activity like many other polyphenols from different sources [33]. Indeed, sesame oil markedly increased NOS activity in the left ventricle and aorta of Zucker fa/fa rats. Furthermore, sesame oil increased significantly the expression of phosphorylated eNOS at 1177 (Ser) in the left ventricle. Addition of simvastatin to this treatment did not lead to further increase of NOS activity and/or expression of phosphorylated eNOS. There were no significant changes in iNOS protein expressions within the groups and tissues investigated. Thus, iNOS expression seems not to participate in increase in total NOS activity.

Hepatic CD concentration was higher in Zucker fa/fa compared with Zucker lean rats, and sesame oil treatment decreased it significantly. Interestingly, this decrease was not seen after sesame oil and simvastatin cotreatment. There were no significant changes in TBARS concentration within the groups; however, decreasing trend in TBARS concentration after SO treatment in Zucker fa/fa rats was seen. In agreement with our study, antioxidant effect of sesame oil and its polyphenolic compounds were documented in atherosclerotic and diabetic conditions [18, 34] or in hyperlipidemic patients [24]. By reducing oxidative load, sesame oil may have direct activated effect on endothelial NOS leading to increase in NO production. We suppose that sesame oil may also increase the stability of NOS cofactor—tetrahydrobiopterin leading to effective NOS activity.

In conclusion, our study clearly documented that neither sesame oil nor an addition of simvastatin affected plasma lipid profile in Zucker fa/fa rats. Sesame oil and similarly sesame oil and simvastatin cotreatment increased NOS activity in the left ventricle and aorta with potential beneficial properties in the cardiovascular system. We suggest that phosphorylation of eNOS and decreased oxidative load may significantly contribute to increase in total NOS activity. Interestingly, simvastatin does not affect nitric oxide generation already increased by sesame oil in obese Zucker rats.

### 4.1. Conclusions

Statins that lower LDL cholesterol and have additional pleiotropic effects are widely used in the treatment of cardiovascular and obesity-related diseases [35–37]. However, there is a considerable residual risk of cardiovascular diseases in patients on statin therapy with some individuals unable to achieve target LDL cholesterol goals even with high doses or are intolerant to the drug [37]. High-dose statin therapy is also occasionally associated with side effects such as locomotion disturbances, nonallergic rhinitis, rhabdomyolysis, and hyperglycemia though some of these are debatable [38]. Substantial research has therefore been carried out on alternative therapies with some recent successes [4]. As documented in our experimental study, by reducing the oxidative load and increasing the activity of protective eNOS, sesame oil seems to be one of these alternatives. Most importantly, sesame oil was able to increase eNOS activity to a level that did not further increase by the addition of simvastatin.

## Figures and Tables

**Table 1:** Lipid profile.

	CHOL	TG	HDL	LDL
Z-lean-C	2.76 ± 0.06^∗^	2.55 ± 0.73^∗^	7.30 ± 0.51^∗^	1.97 ± 0.15^∗^
Z-lean + SO	3.84 ± 0.78^∗^	6.36 ± 3.84	10.33 ± 3.10^∗^	4.23 ± 1.81^∗^
Z-fa/fa-C	6.07 ± 0.13	8.34 ± 0.85	24.55 ± 4.87	10.30 ± 2.76
Z-fa/fa + SO	5.99 ± 0.32	9.76 ± 1.79	18.05 ± 1.08	7.83 ± 1.20
Z-fa/fa + SO + SIM	4.98 ± 0.41	9.63 ± 2.05	14.12 ± 1.35	7.24 ± 1.69

Data are means ± SEM from 6 animals in each group. Z-lean-C: Zucker lean rats; Z-lean + SO: Zucker lean rats treated with sesame oil; Z-fa/fa-C: control Zucker fatty rats; Z-fa/fa + SO: Zucker fatty rats treated with sesame oil; Z-fa/fa + SO + SIM: Zucker fatty rats treated with sesame oil and simvastatin; CHOL: cholesterol; TG: triglycerides; HDL: high-density lipoprotein; LDL: low-density lipoprotein. ^∗^*p* < 0.05 versus Z-fa/fa-C.

## Data Availability

The data used to support the findings of this study are included within the article.
